# Traditional Uses, Phytochemistry and Pharmacological Activities of Annonacae

**DOI:** 10.3390/molecules27113462

**Published:** 2022-05-27

**Authors:** Bassam S. M. Al Kazman, Joanna E. Harnett, Jane R. Hanrahan

**Affiliations:** Faculty of Medicine and Health, The School of Pharmacy, The University of Sydney, Camperdown, NSW 2006, Australia; bassam.alkazman@sydney.edu.au (B.S.M.A.K.); joanna.harnett@sydney.edu.au (J.E.H.)

**Keywords:** Annonaceae, *Annona*, custard apple, phytochemistry, bioactivity, ethnomedicinal pharmacological activity

## Abstract

In 1789, the Annonaceae family was catalogued by de Jussieu. It encompasses tropical and subtropical plants which are widespread in distribution across various continents such as Asia, South and Central America, Australia and Africa. The genus of Annona is one of 120 genera of the Annonaceae family and contains more than 119 species of trees and shrubs. Most species are found in tropical America, where over 105 species have been identified. Due to its edible fruits and medicinal properties, Annona is the most studied genus of Annonaceae family. To date, only a limited number of these species have economic value, including *A. squamosa* L. (sugar apple), *A. cherimola* Mill. (Cherimoya), *A. muricata* L. (guanabana or soursop), *A. atemoya* Mabb. (atemoya), a hybrid between *A. cherimola* and *A. squamosa*, *A. reticulata* L. (custard apple), *A. glabra* L. (pond-apple) and *A. macroprophyllata* Donn. Sm. (ilama). Phytochemically, several classes of secondary metabolites, including acetogenins, essential oils, alkaloids, terpenoids and flavonoids. The pharmacological activities of Annona species leaves and seeds include antibacterial, anticancer, antidiabetic and anti-inflammatory properties.

## 1. Introduction

In 1789, the Annonaceae family was cataloged by de Jussieu [1,2]. It encompasses tropical and subtropical plants, which are widespread in distribution across various continents such as Asia, South and Central America, Australia and Africa [3]. It is one of the largest Mangnoliidae families and the number of its genera and species is still debated [4,5,6]. Bailey and Popenoe believe that it has between 40 and 50 genera and from 500 to 600 species [6]; however, many studies have indicated that the Annonaceae family is comprised of more than 2400 species distributed in approximately 120 genera [4,5]. The family of Annonaceae involves trees, lianas and bushes arranged in four large subfamilies: Malmeoideae, Annonoideae, Ambavioideae and Anaxagoreoideae [7,8]. Economically, species of Annonaceae are important as a source of edible fruits, for instance, the pawpaw (*Asimina*), custard apple, sweetsop, soursop and cherimoya [1]. It has also been reported that some oils from the seeds might be used for the production of edible oils and as an ingredient in soaps, and the woods of some species have been reported for alcohol production [3]. Chemical studies of Annonaceae species have reported the isolation of a wide diversity of phytochemical components, including acetogenins, alkaloids and flavonoids from the bark, fruits, leaves, seeds and pulp of Annonaceae [9]. This review aims to provide a comprehensive summary of the botanical features, phytochemistry, pharmacological properties, and the traditional and ethnomedicinal uses of the Annonaceae family and, specifically, *Annona* species.

## 2. Botanical Features of Annonaceae Species

### 2.1. Distribution and Classification

Annonaceae has been listed as a diverse family of aromatic trees, bushes or shrubs, and climbers or lianas, which are predominantly found in the tropical and subtropical regions, with a limited number growing in temperate zones [1,10]. In tropical America, the Annonaceae species are usually shrubby and most grow in open grasslands [1]. In contrast, species that are climbers mostly grow in the tropical area of the old world [1]. In temperate zones like North America, the only genus reported is Asimina [1,3]. In Brazil, more than 385 species have been reported, with the majority of them reported in the Amazonian region [2]. According to the Takhtajan system of flowering plant classification, the majority of Annonaceae plants can be found in both Asia and Australasia with approximately 51 genera and more than 950 species, while 40 genera with approximately 450 species are confined to Africa and Madagascar, and about 38 genera and 740 species are native to the American continent [3]. The first classification of the Annonaceae family was described by Dunal in 1817 and was limited to only fruit morphology [11]. Subsequently, a new classification of the Annonaceae family based on flower characteristics was introduced by Diels and Alder in 1932 [11]. However, a later classification by Fries in 1959 was found to be more comprehensive and authentic, using a combination of fruit morphology and flora characteristics [11]. The Annonaceae family are characterised by the presence of a variety of primitive and archaic features, leading to them being described by Darwin as “living fossil” due to their ability to survive the mass extinction [1,11]. Under the Takhtajan system, the Annonaceae family is related to Magnoliaceae, which is one of the largest families of Magnoliales with other families such as Degeneriaceae, Canellaceae, Himantandraceae and Myristicaceae [1,11].

### 2.2. Diagnostic Features

From one species to another, the botanical features of Annonaceae families vary greatly based on their origin, geography, and climate. Based on morphology and habitat, the Annonaceae family is known among the homogeneous plant families [1,4]. The aromatic flowers are commonly open before other parts are entirely developed. The flowers are terminal, axillary, hermaphrodite, singular or grouped and regular [1,11]. The stamens are typically abundant, spirally arranged and hypogenous [1,11]. The leaves are characterised by having a glaucous or metallic sheen, and they are alternate, exstipulate and regular [1,11]. The fruits are typically made up of clusters of berries with an edible fleshy receptacle, particularly in the *Annona* genera and they are extensively consumed due to their high nutritional value [1,11]. Finally, the seeds are enlarged and have a copious, irregular-surfaced endosperm with a minute embryo [1,11].

### 2.3. Traditional Uses

Annonaceae species are famous in tropical regions and used traditionally across tropical regions due to their widespread distribution. Various parts of the species are used traditionally, including leaves, seeds, bark, fruit, stem, roots and twigs. A range of different methods for preparation is reported, such as infusions, pastes and decoctions [11]. For instance, the fresh fruit of *Annona dioica* is used for wound healing in Brazil [11]. The dried leaves of *Annona muricata* are used orally for analgesic effects in some parts of Indonesia [11]. In Burkina-Faso, the bark and roots of *Annona muricata* are used for dysentery and as an anthelmintic medicine, whereas the leaves are utilized for both fever and dysentery [12]. In the northwestern part of Brazil, both leaves and twigs of *Duguetia chrysocarpa* are ground and the extract of this mixture are utilized for treating gastrointestinal ulcers as well as a remedy for bowel disease [11]. A decoction of the stem bark of *Annickia chlorantha* is used orally as a remedy for the treatment of wounds and fever in Cameroon [13]. Further data on the traditional uses of the most widely used Annonaceae species are presented in Table 1.

## 3. Phytochemistry of Annonaceae Family

A wide array of chemical compounds from various parts of Annonaceae plants have been discovered, isolated and characterised. The results of both phytochemical investigations and biological studies on various plants from this family have led to the identification of a wide diversity of compounds such as annonaceous acetogenins, flavonoids, alkaloids and essential oils, as summarized in (Table 2). These phytochemical constituents have been found to exhibit a broad range of biological activities such as immunosuppressive, antineoplastic, cytotoxic, antimicrobial, anti-inflammatory effects (Table 3). However, it is the *Annona* genera that are the most widely used as a food source and in traditional medicines.

## 4. *Annona* Genera

The genus of *Annona* is one of the 120 genera of the Annonaceae family and contains more than 119 species of trees and shrubs, most of them distributed in tropical areas of the Americas and Africa [6]. The majority of these species are found in tropical America, with more than 105 species (26 of them are endemic) and 10 species distributed in tropical Africa [10,34]. It has been reported that this genus is the second or the third largest genus in the Annonaceae family [35]. Its generic name derives from the Latin Hispaniolan Taino “annual harvest” [6,35]. Due to its edible fruits and medicinal properties, Annona is the most important genus of Annonaceae family [2]. Numerous Annona species furnish edible fruits like *Annona muricata* (“graviola”), *Annona crassiflora* (“araticum”) and *Annona sqaumosa* (“fruta do conde”) [2]. Most of the fruits are consumed either in fresh form or used in desserts, juices and ice cream preparations [34]. Despite Annona having many species, only limited species of this family are economically important such as *A. squamosa* L. (sugar apple), *A. cherimola* Mill. (Cherimoya), *A. muricata* L. (guanabana or soursop), *A. atemoya* Mabb. (atemoya), a hybrid between *A. cherimola* and *A. squamosa*, *A. reticulata* L. (custard apple), *A. glabra* L. (pond-apple) and *A. macroprophyllata* Donn. Sm. (ilama) [6]. Phytochemically, several classes of secondary metabolites such as acetogenins, essential oils, alkaloids, terpenoids and flavonoids have been described in this genus [34,36]. A variety of pharmacological activities have been reported from various parts of Annona species specially leaves and seeds including applications against antibacterial [37], antinociceptive [38], anticancer [39], anticonvulsant [40], antidiarrhea [41], antidiabetic [42], antimalarial [39], anti-inflammatory [43], antioxidant [44], antileishmanial [45], antiulcer [46] and antidepressant [47].

### 4.1. Botanical Features

Generally, *Annona* species are small trees or shrubs with a height from 5 to 11 m depending on various factors including soil, climate, species, and crop management [2]. In relation to the botanical characteristics of *Annona* species, the majority of them are moderately erect with brown bark that is frequently furrowed (Table 4) [10]. The stems are rust-coloured (ferruginous) and covered with densely matted hairs (tomentose) when young, becoming smooth and hairless (glabrous) as they mature [6,10]. It has thin lateral roots and a taproot that is not generally pronounced [2]. With regard to the flowers, they are hermaphrodites, solitary or fascicle containing from two to four flowers. The flowers are usually fragrant, with six petals and three green sepals, in a circular arrangement of two verticils [6]. Flowering of the plant usually starts at 3 to 4 years and flower opening usually occurs by separation of the apex of external petals [6,10]. Finally, the leaves may be shiny or hairy and have an impressed vein on the upper side, and the fruits are syncarpous and comprised of seeds and many carpels [6,10].

#### 4.1.1. *Annona* Cherimola

*Annona cherimola Mill (Cherimoya)* belongs to the genus *Annona* in the Annonaceae family in magnolias order, which means “cold seeds”, and is a small tree that produces heart-shaped and conical edible fruit [55]. It is a steep, semi-momentary and a low bunched tree that is widespread in Ecuador and Peru and distributed throughout Asia, South Europe, America and Africa [56]. In Mexican traditional medicine, this plant has been used to treat various diseases such as diabetes, cough, fever, headache, worms and inflammation either alone or in combination with other plant species [48,57,58,59,60]. Recently, various parts of *A. cherimola* have been phytochemically profiled and contain various polyphenols and alkaloids. The leaves were found to be a source of bioactive compounds with potential for use as treatments for skin and eye diseases and gastric, cardiovascular and intestinal disorders [55].

#### 4.1.2. *Annona Squamosa*

*Annon squamosa* L., commonly known as custard apple, is a tropical, endemic species of the West Indies, Ecuador, Peru, Brazil, South and Central America, Mexico, Bahamas, Bermuda, and Egypt [61]. This plant is extensively cultivated in various states of India, including Maharashtra, Gujarat, Madhya Pradesh, Chhattisgarh, Assam, Uttar Pradesh, Bihar, Rajasthan, Andhra Pradesh, and Tamil Nadu. The total area of cultivation has been reported by the Indian Council of Agricultural Research (ICAR) as 40,000 ha [62]. Its tree grows as a small sapling from 3 m to 8 m, with large branches having brownish or light brownish bark and it has thin leaves and is known for its edible fruit [61]. In the Aligarh district village in Uttar Pradesh, *A. squamosa* is well-known for its antidiabetic properties [63]. Its seeds, bark and leaves possess various pharmacological properties, mainly anti-tumour properties [64].

#### 4.1.3. *Annona Muricata*

*Annona muricata* is a commonly known as soursop and graviola and is native to Central and South America. It is a small tree 5–10 m tall and 15–83 cm in diameter; it has low branches and edible fruit that are used commercially for the production of candy, juice and sherbets [65]. Traditionally, the aerial parts of this plant have been used to treat various diseases like diabetes and malaria and nowadays, it is widely used by people diagnosed with cancer [66]. Moreover, this species possesses several pharmacological properties, including vasodilator, cardio-depressive, antispasmodic, antimutagen, anticonvulsant, antiviral, antidiabetic and antihypertensive effects [67]. Both the leaves and seeds of *A. muricata* have been evaluated for their constituents resulting in the identification and isolation of more than 50 mono-THF acetogenins, alkaloids, terpenoids, saponins, flavonoids, coumarins, cardiac glycosides, phenols, tannins and anthraquinones [67].

#### 4.1.4. *Annona Reticulata*

*Annona reticulata* Linn is a traditionally important plant utilized in traditional medicines [68]. It is indigenous to the West Indies and widely distributed in tropical and subtropical regions of the world [54]. It is a small tree with a height between 6 and 7.5 m and contains numerous lateral branches [54]. It has a cylindrical stem that contains lenticels and very short coffee-colored hairs [54]. The leaves of *A. reticulata* are lanceolate, membranous, oblong, and rounded or curate at the base. Fruits are edible, rough, somewhat heart-shaped and yellow in color that shifts to yellowish-red on ripening, and the seed is smooth and blackish in color [69]. Traditionally, *A. reticulata* has been utilized for the treatment of epilepsy, dysentery, cardiac problem, constipation, haemorrhage, bacterial infection, parasite and worm infestations, fever, ulcers and as an insecticide [68,69]. Its leaves are used for helminthiasis treatment while bark is a powerful astringent and used as a tonic [68,69].

#### 4.1.5. *Annona Coriacea*

*Annona coriacea* Mart. is a species belonging to the *Annona* genera, commonly known as “marolo”, “araticum” and “araticum-liso” [70]. This plant is distributed across Paraguay and Brazil, with little available information about its ethnomedicinal uses [71]. It is a small tree (3–6 m) and its edible fruit consist of an ovoid-obtuse syncarp and weighing up to 1.5 kg [72]. The leaves are glabrous on the ventral surface, obovate, and the base is frequently cordate and margin undulate [73]. The flowers are terminal, thick, solitary and having fleshy petals with colors shifting between orange and pink [73]. The leaves are traditionally used as carminatives, anthelmintics, antirheumatics and in the treatment of stomatitis, headaches, abscesses, neuralgia, rheumatism, ulcers and dermatitis [74,75]. Both seeds and fruits are toxic when crushed and exhibited effects against ectoparasites like lice [75].

#### 4.1.6. *Annona Senegalensis*

*Annona senegalensis* is a small tree 2–6 m tall that is commonly known as wild custard apple and wild soursop [76]. This plant is native to tropical east and northeast, west and west-central, and southern Africa and islands in the western Indian Ocean [76]. Its leaves are simple, alternate, oblong, green to bluish-green, ovate or elliptic, and mainly lack hairs on the upper surface and brownish hairs on the lower surface [77]. This plant has been used in traditional medicine as a pain reliever, antioxidant, antidiarrheal, antitrypanosomal, antimalarial, anti-inflammatory, antimicrobial, antiparasitic, anticonvulsant and as an anti-snake venom [78]. It has been reported that the leaves of *A. senegalensis* are used for the treatment of tuberculosis, yellow fever and smallpox, whereas stem bark is reported for the treatment of injury from venomous animals [79]. The root was also reported for treating erectile dysfunction, tuberculosis, gastritis, reproductive deficiency and in the management of malaria and diabetes [80].

#### 4.1.7. *Annona Vepretorum* Mart

*Annona vepretorum* is commonly recognized as ‘bruteira’, is a small tree of 2.5–10 m high native to the Brazilian biome Caatinga [81]. The fruits of *A. vepretorum* can be consumed either raw or as juice for nutritional purposes [82]. Traditionally, a decoction of the leaves have been used to bathe in for the treatment of allergies, yeast, skin diseases and microbial infections, whereas the root is traditionally used to treat snake and bee bites, inflammatory conditions and heart pain [81].

#### 4.1.8. *Annona Salzmannii*

*Annona salzmannii* is a tree of 6–20 m high that known as “araticum-da-mata” and “araticum“apé” [83]. It is commonly cultivated in Brazil especially in the States of Bahia, Pernambuco, and Paraíba [83]. Its root, seeds and leaves are used in folk medicine for treating several illnesses like ulcers, dysentery and inflammatory conditions [83]. The leaves and bark of *A. salzmannii* are utilized for the treatment of tumors, diabetes and inflammatory conditions [84].

#### 4.1.9. *Annona Crassiflor*

*Annona crassiflor* is known as araticum of cerrado or cerradão [85]. It is a small tree that bears a typical fruit known as araticum of cerrado or cerradão [86]. The fruits are highly consumed “in natura” by native people and can be used to make juice, jelly and ice-cream [85,86]. In folk medicine, the seeds are used to treat scalp infections, and infusions of the leaves and seeds are utilized for their antidiarrheal and antitumor properties [85]. For more details about the botanical characteristics and traditional uses of *Annona* species, see Table 4 and Table 5.

### 4.2. Traditional and Ethnomedicinal Uses of Annona Genus

Traditionally, the *Annona* species have been used widely. For instance, antidiarrheal effects have been reported for *A. reticulata*, *A. muricata* and *A. salzmannii*, whereas *A. cherimola*, *A. squamosa* and *A. reticulata* have been reported for their antiparasitic effects (Table 5) [10]. Moreover, both *A. vepretorum* and *A. salzmannii* have been also reported for anti-inflammatory effects [10]. *A. purpurea and A. reticulata* have been used to treat fever, while anticancer effects have been reported for *A. senegalensis* and *A. muricata* [105,106]. Furthermore, *A. foetida*, *A. muricata* and *A. glabra* have been traditionally used to treat rheumatism [107], while *A. reticulata*, *A. salzmannii*, *A. foetida* and *A. squamosa* have been described for treating ulcers [4]. In Indonesia, the fruit juice of *A. muricata* has used as a diuretic and to treat liver ailments and leprosy [108], whereas leaves was used to treat spasms, boils and as an aphrodisiac [36]. The leaves of *A. diversifolia* have been used as anti-inflammatory, anticonvulsant and analgesic agents [52]. Ethnobotanically, despite reports of the toxicity of *A. muricata* seeds, the powder of toasted seeds has been reported to be used as an emetic and cathartic in the traditional Mexican pharmacopeia [36]. To Southeast Asian people, the immature fruit of *A. reticulata* was used to treat both dysentery and diarrhea, and a decoction of roots was used to cure toothache and as an antipyretic [109]. Additionally, a decoction of leaves has been used internally against worms and topically to treat abscesses and boils [109]. Finally, the leaves of *A. squamosa* have been used as tonic and cold remedy in tropical America and systemically to cure dysentery in India [108].

## 5. Phytochemistry of *Annona* Species

**Figure 1:**
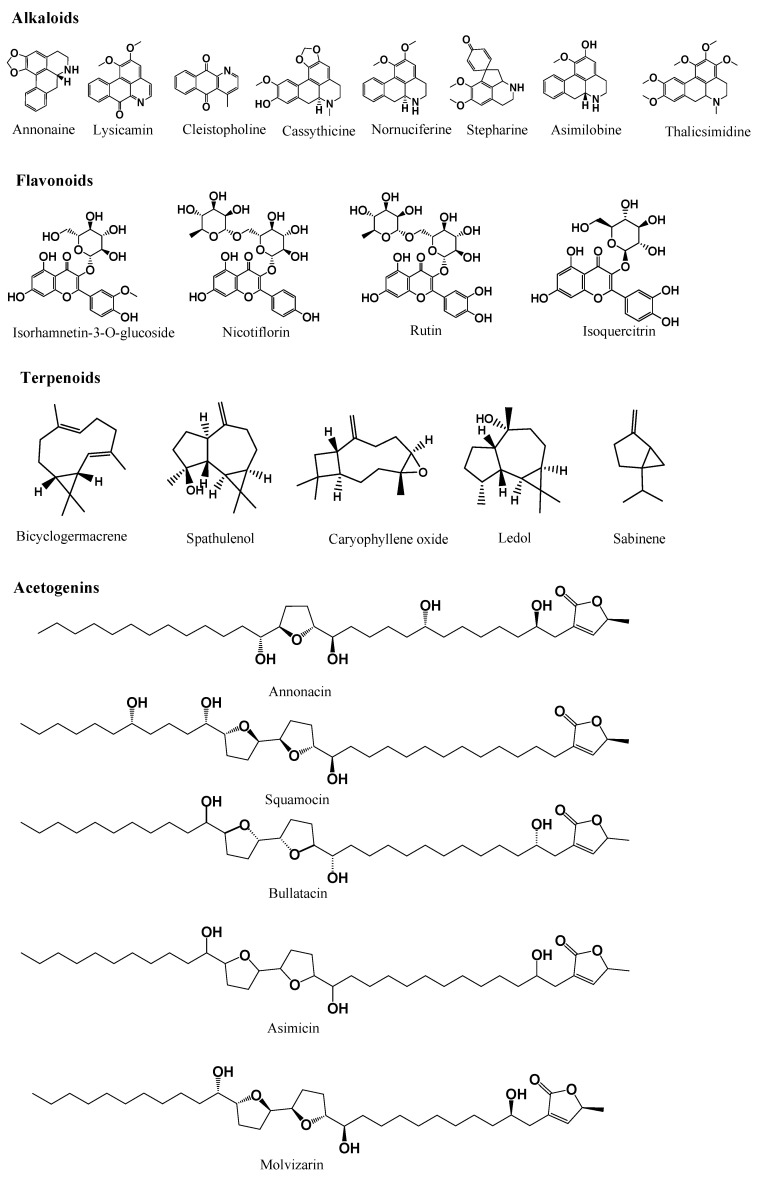
Structure of selected compounds identified in Annona species.

A wide range of secondary metabolites, including acetogenins, flavonoids, alkaloids and essential oils (Figure 1), from nearly every part of *Annona* plants, have been discovered, isolated and characterised (Table 6). The plants of the *Annona* genera are also found to be rich in minerals and vitamins, for instance, calcium, potassium, magnesium, sodium, copper, zinc, selenium, phosphorus, iron, vitamin C, pantothenic acid B_5_, thiamine and riboflavin [6].

## 6. Pharmacological Properties of *Annona* Species

The species of the *Annona* genera have a been reported to elicit a diversity of biological activities such as antitumor, anti-inflammatory, antioxidant, antinociceptive, antiprotozoal, antipyretic, antiulcer, antihyperglycemic, anthelmintic, antileishmanial, antimalarial, antidiarrheal, antifungal and antimicrobial promoted by whole extracts, fractions, or pure compounds (Table 7).

### 6.1. Antibacterial Activity

The antibacterial activities of *Annona* species have been reported in many studies, for example, both methanolic and ethanolic leaf extracts of *A. muricata* exhibited antimicrobial activity against *Staphylococcus aureus,* and this activity was attributed due to the presence of flavonoids, alkaloids and steroids in the extract [165,166]. In contrast, an aqueous extract of the peel of *A. muricata* did not show any activity [165,166]. The root of *A. reticulata* has also been investigated for its antibacterial activity against Gram-positive *Staphylococcus aureus, Bacillus cereus and Bacillus subtilis*, and Gram-negative *Pseudomonas aeruginosa, Escherichia coli* and *Salmonella typhi* [54]. The root extract was found to possess pronounced activity against *Bacillus cereus* as well as notable inhibition against all tested strains [54]. Moreover, the leaves of *A. cherimola* have been reported for antibacterial activity against *Staphylococcus aureus* and *Bacillus subtilis* with growth inhibition zone diameters of 11 mm and 14 mm, respectively [37]. The aqueous and methanolic seed extracts of *A. squamosa* have reported activity against *Staphylococcus aureus with* Minimum Inhibitory Concentrations (MIC) of 50 mg/mL and Minimum Bactericidal Concentrations (MBC) of 100 mg/mL [167]. The activity of the isolated compounds from *Annona* species has been reported in various studies; for instance, the fatty alcohol 11-hydroxy-16-hentriacontanone isolated from leaves of *A. squamosa* has a reported activity against Gram-positive and Gram-negative bacterial strains, with MIC values of 25–50 µg/mL [168]. Additionally, the alkaloids liriodenine, annonaine, asimilobine, reticuline and cleistopholine isolated from *A. salzmannii* demonstrated activity against a range of Gram-positive bacteria, including *Kocuria rhizophila, Staphylococcus aureus, Staphylococcus epidermidis and Enterococcus faecalis* with MIC values from 25 to 500 µg/mL [36]. Notably, annonaine and asimilobine had activity equal to or better than the control chloramphenicol (MIC 50 µg/mL) against many of the species tested [36]. 

### 6.2. Anticancer and Antiproliferative Activity

Various studies have reported the anticancer activity of either crude extracts or isolated compounds from *Annona* species. For example, the leaves extract of both *A. squamosa* and *A. reticulata* exhibited potent antiproliferative effects against two human T-lymphotropic virus type = 1 infected cell lines (MT-1 and MT-2) with EC50 values from 0.1 to 1 µg/mL [169]. In in vitro studies, the ethanolic extract of A. muricata leaves was reported for its cytotoxicity against promonocytic leukemic cells (U-937) with an LC_50_ = 7.8 µg/mL [170]. Isocoreximine isolated from *A. cherimola* demonstrated cytotoxicity against multiple cancer cell lines. At a concentration of 50 µg/mL, isocoreximine inhibited cell viability of the breast cancer cell line (MCF-7) by 85.76%, human colorectal carcinoma cell line (HCT-15) by 63.05%, human prostate tumor cell line (PC-3) by 78.71%, human astrocytoma cell line (U-251) by 65.23% and human leukemia cell line (K-562) by 94.15% [112].

The antiproliferative activity of methanolic extracts from the leaves and seeds of *A. coriacea* was tested in vitro against a range of human tumor cell lines; including melanoma (UACC-62), non-small cell lung cancer cells (NCI-H460), colon cancer cell line (HT29), breast cancer (MCF-7) and leukemia (K-562). The seed extracts displayed potent antitumor activity with GI50 values between 0.02 and 3.83 µg/mL, and the leaf extracts exhibited anticancer activity at concentrations ranging from 0.02 to 0.08 µg/mL [35,171]. The cytotoxicity of annonacin, found in many *Annona* species, has been reported against various cell lines derived from cervical cancer (HeLa and HeLa S3] with IC_50_ 0.219 and 0.426 µg/mL, and ovarian cancer (PA-1 and SKOV3) with IC_50_s of 0.452 and 0.411 µg/mL [172]. The cytotoxicity of annonacin has also been demonstrated against bladder cancer (T24), breast cancer (MCH7) and skin cancer (BCC-1) with IC_50_s 0.324, 0.433 and 0.427 µg/mL, respectively [172]. The cytotoxicity of five other acetogenins (squamocin M, annofolin, isolongimicin B, glaucanisin, and annotacin) isolated from *A. cornifolia* against human breast cancer (MCF-7) was reported, with IC_50_s of approximately 0.3 µM [173]. In an in vitro study, the annoaceous acetogenins laherradurin and cherimolin-2 isolated from *A. diversifolia* were shown to have ED_50_s of 0.015 and 0.05 µg/mL, respectively, against the cervical cancer cell line (HeLa) [174].

In clinical studies, the anticancer activity of *A. muricata* has been reported in a small number of studies. A patient diagnosed with breast cancer has maintained stable disease activity with no reported side effects after using an aqueous extract of *A. muricata* leaves for more than five years [175]. Another patient with metastatic ovarian cancer experienced disease stability after starting to take a complementary medication containing *A. muricata* as a tablet [176]. Finally, the effect of *A. muricata* leaves extract revealed higher cytotoxicity in the supplemented group with colorectal cancer compared with the placebo group in a randomized controlled trial [177].

### 6.3. Antidabetic and Antilipidemic Activity

Multiple studies have investigated the antidiabetic activity of various extracts from *Annona* plants such as *A. cherimola*, *A. squamosa*, *A. muricata* and *A. reticulata*. The ethanolic leaf extract of *A. cherimola* (300 mg/kg) was administered to alloxan-induced type 2 diabetic rats, and four hours later, blood glucose level had decreased from 331.5 mg/dL to 149.2 mg/dL [58]. The young leaves of *A. squamosa,* often in combination with black pepper (*Piper nigrum*), have been used in northern Indian traditional medicine as an anti-diabetic, and are still in use today. Administration of aqueous *A. squamosa* leaf extract to streptozotocin-nicotinamide type-2 diabetic rats resulted in decreased blood glucose and increased levels of serum insulin [178]. Another traditional Indian medicine used as an antidiabetic and anti-lipidemic is a polyherbal formulation of *A. squamosa* fruits and *Nigella sativa* seeds. The polyherbal formulation administered over a one-month period, dose-dependently decreased blood glucose and increased insulin in streptozotocin-induced diabetic rats, with a dose of 200 mg/kg showing similar to the effects of a dose of 250 mg/kg tolbutamide [179]. A single dose of 100 or 200 mg/kg of aqueous leaf extract of *A. muricata* did not inhibit blood glucose levels in normal rats; however, the same doses administered to the diabetic rats effectively lowered blood glucose levels by 31.77% and 45.77%, respectively [180]. Finally, a dose of 100 mg/kg of both methanolic extract and the residual fractions of *A. reticulata* leaves decreased blood glucose levels from 432.33 to 371.67 mg/dL and 417.83 to 402.50 mg/dL, respectively, in streptozotocin-induced diabetic rats [181]. *A. cherimola* leaf extract was also found to decrease HbA1c by 7% and lead to a significant decrease in urine glucose over a 28-day subchronic study in streptozotocin-induced diabetic mice [182]. These studies support the traditional use of *Annona* species as antidiabetics, suggesting that further identification of the active constituent(s) with antidiabetic properties and clinical studies of a longer duration are warranted.

Limited studies have also investigated the antilipidemic activity of some *Annona* species. The polyherbal formulation of *A. squamosa* fruits and *Nigella sativa* seeds (200 mg/kg) administered to streptozotocin-induced diabetic rats for one month also resulted in significant inhibition of the formation of both lipid peroxide and tissue lipids [179]. Administration of an extract of *A. muricata* extracts resulted in reductions in the serum total cholesterol, low-density lipoprotein cholesterol and triglycerides in diabetic rats [183]. The tea infusion of leaves from *A. cherimola* (1.5 g) also elicited a reduction in the total cholesterol, triglycerides, and low-density lipoprotein by 15.4, 21.9 and 63.2%, respectively, in streptozotocin-induced diabetic rats [182].

### 6.4. Anti-Inflammatory Activity

The anti-inflammatory effects of *Annona* plants have been reported in many studies; for instance, after one day of orally administered doses of 200 and 400 mg/kg of *A. squamosa* root extract in an acute carrageenan-induced rat paw edema model, significant inhibition was produced with 24% and 47% inhibition respectively compared to diclofenac sodium inhibiting inflammation by 72% [184]. In an in vitro study, the chloroform extract of *A. muricata* leaves significantly inhibited activity of phospholipase A_2_ [185]. With doses of 0.2–0.6 mg/mL, the enzyme activity was inhibited by 23.91%–43.48% [185]. In the same study, the chloroform extract of *A. muricata* leaves at 0.5 and 1.0 mg/mL also inhibited prostaglandin synthase activity by 87.46% and 82.92%, respectively, compared to the positive control indomethacin at 1 mg/mL which reduced enzyme activity by 87.46% [185].

Extracts of *A. senegalensis* roots were assessed for anti-inflammatory activity through in vitro inhibition of protein denaturation, hyaluronidase and xanthine oxidase. The ethyl acetate fraction was found to have the greatest activity inhibiting protein denaturation (70.6%), hyaluronidase (72.2%) and xanthine oxidase (78.7%) at a concentration of 100 µg/mL [186]. The ethanolic extract of *A. muricata* leaves exhibited anti-inflammatory effects in the carrageenan-induced rat paw acute edema model. Paw edema was reduced after orally administered doses of 200 and 400 mg/kg with (23.16% and 29.33%) and (29.50% and 37.33%), respectively, after 60 and 90 min of treatment [187]. Additionally, *A. muricata* fruit has been shown to exert an anti-inflammatory effect in a xylene-induced ear edema test [188]. Additional information regards pharmacological activities of *Annona* species, see (Table 7).

The lyophilized fruit extract at 50 mg/kg and 100 mg/kg inhibited the xylene-induced ear edema by 82.35% and 76.47%, respectively, compared to prednisolone, which reduced ear edema by 47.06% [188]. At intraperitoneal doses of 25, 50 and 100 mg/kg, the ethanolic extract of *A. vepretorum* leaves, inhibited carrageenan-induced leukocyte migration to the peritoneal cavity by 62%, 76% and 98%, compared to dexamethasone (2 mg/kg, i.p.). which reduced leukocyte migration by 89% [104]. The flavonoids, quercetin and kaempferol isolated from leaves of *A. dioica* exhibited potent dose- and time-dependent anti-inflammatory activity in a carrageenan-induced paw oedema model with IC_50_s of 8.53 and 10.57 µg/mL, respectively. The crude methanolic extract of *A. dioica* also reduced myeloperoxidase activity 6 h after the induction of paw oedema with a maximal inhibition of 51% at a dose of 300 mg/kg. [189]. Finally, hinesol, z-caryophyllene and beta-maaliene isolated from leaves of *A. sylvatica* also inhibited leukocyte migration at concentrations from 36.04 to 45.37 µg/mL in both carrageenan- and complete Freund’s adjuvant-induced mouse paw edema [33].

### 6.5. Antioxidant Activity

An extract of *A. coriacea* seeds was investigated for its antioxidant activity using free radical 2,2-diphenyl-1-picrilhidrazil (DPPH) and bleaching of β-carotene, and a moderate antioxidant effect was reported of 31.53% in the DPPH test and 51.59% for the β-carotene bleaching test [190]. The pulp of *A. coriacea* fruit displayed a weaker antioxidant activity compared to the seeds, with 13.49% for the DPPH test and 32.32% for the β-carotene assay [190]. Additionally, various parts of *A. muricata,* including bark, leaves and stem, exhibited antioxidant activity using DPPH assay and the EC_50_ value was recorded as 90 mg/g for bark, 290 mg/g for leaves, and 116 mg/g for the stem, compared to ascorbic acid with 157.5 mg/g [44]. Finally, an ethanolic extract of *A. squamosa* leaves was also reported as having antioxidant activity when evaluating DPPH, nitric oxide and superoxide radical assays. The activity was reported as 75.12%, 34.69% and 10.29%, respectively, at a concentration of 100 µg/mL [191].

### 6.6. Antileishmanial Activity

Many extracts and pure compounds from *Annona* plants have been tested against *Leishmania,* such as methanolic seed and leaf extracts of *A. squamosa,* for activity against *L. amazonensis,* with resulting showing IC_50_s of 46.54 µg/mL and 28.32 µg/mL, respectively [192]. Alkaloids and acetogenins isolated from both leaves and seeds of *A. squamosa* were also reported for their activity against promastigote forms of *L. chagasi,* with the EC_50_ value reported as 23.3 µg/mL for alkaloids and from 25.9–37.6 µg/mL for acetogenins [192]. Alkaloids like liriodenin isolated from the leaves of *A. mucosa* exhibited antileishmanial activity against promastigote forms of *L. braziliensis, L. guyanensis* and *L. amazonensis* with IC_50_s of 55.92 μg/mL, 0.84 μg/mL 1.43 μg/mL respectively [193]. Finally, *O*-methylarmepavine isolated from leaves *A. squamosa* displayed antileishmanial activity against both promastigote and amastigote forms of *L. chagasi* with EC_50_s of 23.3 μg/mL and 25.3 μg/mL, respectively [45].

### 6.7. Antiviral Activity

The antiviral activity of various *Annona* species was reported in several studies using either whole extract or pure compounds. For instance, 16*ß*,17-dihydroxy-*ent*-kauran-19-oic acid was isolated from the fruits of *A. squamosa* and showed significant activity against human immunodeficiency virus (HIV) replication using H9 lymphocyte cell assay with EC_50_ value of 0.8 μg/mL [194]. The ethanolic extract of *A. squamosa* seeds at 0.15 μg/mL, 0.25 μg/mL and 0.35 μg/mL also exhibited dose-dependent antiviral activity against the Avian influenza virus with the percentage of antiviral activity at 33.33%, 43.06% and 59.72%, respectively [195]. The leaves of *A. squamosa* extract were also tested against dengue virus type-2 (DENV-2) in Vero cells using Viral ToxGLo^TM^ assay. At a concentration of 6.25 μg/mL, DENV-2 replication was reduced with IC_50_ 73.78 μg/mL in Vero cells [196]. The methanolic extracts from the peels of *A. squamosa* and *A. reticulata* demonstrated antiviral activity against human immunodeficiency virus 1 (HIV-1) using a non-radioactive immune/colorimetric assay. Both *A. squamosa* and *A. reticulata* revealed high antiviral activity by inhibition of HIV-1 reverse transcriptase with values of 96.45% and 78.63% [197]. Moreover, *A. cherimola* was also evaluated for its antiviral activity against herpes simplex virus type 2 (HSV-2) using 3-(4,5-dimethylthiazol-2-yl)-2,5-diphenyl-2H-tetrazolium bromide (MTT) assay. The leaf extract inhibited HSV-2 replication and showed antiherpetic activity with a therapeutic index 8.40 [198]. Finally, the ethanolic stem extraction of *A. muricata* demonstrated antiviral activity against herpes simplex virus type 1 (HSV-1) with a minimum inhibitory concentration (MIC) of 1 mg/mL [199].

## 7. Pharmacological Activity of Isolated Compounds from *Annona* Species and their Mechanism of Action

The in vitro and in vivo biological activity of compounds that have been isolated from various parts of *Annona* plants will be discussed. Squamins C–F were isolated from the seeds of *A. globifora* and tested in vitro against trophozoites of *Acanthamoeba* spp. strains such as *A. castellanii* Neff, *A. polyphaga*, *A. griffin* and *A. quina* (Table 8) [200]. All tested compounds exhibited antiamoeboid activity against the strains by inducing programmed cell death [200]. The same compounds were also tested for their cytotoxicity against murine macrophage cell line J774A.1 (ATCCTIB-67) and showed no cytotoxicity effect with CC_50_ values greater than 200 μM [200].

Rollinicin and rolliniastatin-1 isolated from the seed of *A. mucosa* were also reported for their larvicidal effect against *Aedes aegypti* and *Aedes albopictus* larvae [201]. Rolliniastatin-1 exhibited the best larvicidal effect against both *Aedes aegypti* and *Aedes albopictus* with LC_50_s of 0.43 and 0.20 μg/mL^−1^, respectively. Rollinicin displayed similar activity against *Aedes aegypti* and *Aedes albopictus* with LC_50_s of 0.78 μg/mL and 1.128 μg/mL, respectively [201]. However, the larvicidal mechanism action of these compounds was not reported. Annonacin isolated from the seed of *A. muricata* was evaluated for its larvicidal activity on *Aedes aegypti* and *Aedes albopictus* larvae [202]. The greater larvicidal activity was reported against *Aedes aegypti* with a LC_50_ of 2.65 μg/mL compared to *Aedes albopictus* with LC_50_ of 8.34 μg/mL. The mechanism of action was reported as being inhibition of their metabolic enzymes, particularly proteases and amylases that are important for the development of *Aedes* spp. larvae [202]. Twelve acetogenins isolated from the seed of *A. cornifolia* were tested for their antioxidant activity against DPPH [90]. These acetogenins were identified as 9-hydroxyfolianain, 4-desoxylongimicin, squamocin M, squamocin L, folianin A, folianin B, annofolin, isolongimicina B, bullatacin, asimicin, cornifolin and anotacin, and showed a strong DPPH radical scavenging with IC_50_s ranging from 0.99 ± 0.18 to 1.95 ± 0.34 μg/mL compared to ascorbic acid with an IC_50_ 1.62 ± 0.35 μg/mL [90]. It has been suggested that the antiradical activity of acetogenins may be related to the α,β-unsaturated lactone ring moiety, which is also present in ascorbic acid [90]. Furthermore, the antioxidant activity of pure compounds from the bark of *A. salzmanni* after isolation of five alkaloids identified as liriodenine, anonaine, asimilobine, reticuline and cleistopholine [203]. The antioxidant activity was assessed through the Oxygen Radical Absorbance Capacity (ORAC) assay and asimilobine was found to be the most active alkaloid with ORAC value of 2.09 [203]. The rest of the compounds exhibited antioxidant activity with ORAC values ranging from 0.25 to 0.85 [203]. These same compounds were also examined for their antimicrobial activity against *Kocuria rhizophila, Staphylococcus aureus, Staphylococcus epidermidis and Enterococcus faecalis* with MIC values from 25 to 100 μg/mL [203]. In an in vitro study, the antimicrobial activity of isolated alkaloids from aerial parts of *A. senegalensis* was assessed in a microdilution assay [154]. These alkaloids were identified as anonaine and asimilobine, and demonstrated against *Streptococcus mutans* with MIC values of 0.12 and 0.25 mg/mL, respectively [154]. For trypanocidal activity, three alkaloids liriodenine, annomontine, and *O*-methylmoschatoline were isolated from the branch of *A. foetida* and tested against both epimastigote and trypomastigote forms of *Trypanosoma cruzi* [101]. A potent trypanocidal effect was demonstrated against epimastigote forms with IC_50_s ranging from 92.0 ± 18.4 to 198.0 ± 4.2 μg/mL, and from 3.8 ± 1.8 to 4.2 ± 1.9 μg/mL for trypomastigote forms [101]. Additionally, *N*-hydroxyannomontine isolated from the bark of *A. foetida,* demonstrated antileishmanial activity versus *Leishmania. braziliensis* and *L. guyanensis* with IC_50_ values of 252.7 ± 2.2 and 437.5 ± 2.5 μM, respectively [204].

Many compounds isolated from various *Annona* species have demonstrated cytotoxicity against different cancer cell lines (Table 9). Three alkaloids were isolated from leaves of *A. crassiflora* identified as crassiflorine, xylopine and stephalagine and tested for their activity against colon carcinoma cells (HCT-116) using MTT assay [204]. The cytotoxicity activity for the tested compounds was reported with IC_50_ values of 143.4 μM, 30.2 μM and 48.5 μM, respectively [204]. Muricin J–K isolated from the fruit of *A. muricata* exhibited anticancer activity against human prostate cancer cell lines (PC-3) through inhibition of the mitochondrial complex I in an in vitro study [205]. The anticancer activity was also reported for the alkaloid coclaurin isolated from aerial parts of *A. squamosa*. Cytotoxicity studies against human breast cancer cells (MCF-7), human colon cancer cells (HCT116) and human liver cancer cells (HEPG-2) reported IC_50_ values of 15.345 μg/mL, 8.233 μg/mL and 1.674 μg/mL, respectively [206]. Bullatacin isolated from *A. cherimola* demonstrated inhibition of tumor growth at a dose of 15 μg/kg in mice bearing HepS and S180 xenografts and tumor growth was reduced by 63.4% and 65.8%, respectively [207]. In the same study, annonacin administrated orally (10 mg/kg) to hybrid mice (BDF-1) models significantly reduced lung cancer by 57.9% [207]. However, the mechanism of action of these acetogenins was not described in this study. Finally, stephalagin, an alkaloid isolated from the peel of *A. crassiflora* fruit, was reported as pancreatic lipase inhibitor with an IC_50_ of 8.35 μg/mL^−1^ in vitro study [208].

## 8. Toxicity and Interactions

Generally, the safety of natural medicines can be assessed according to their effects and drug-drug interactions. An epidemiological study has reported that consumption of fruits of Annonaceae led to the prevalence of atypical parkinsonism in Guadeloupe due to the presence of acetogenins in the plant fruits [238]. According to Champy et al. 2005, the amount of annonacin per a single fruit is approximately 15 mg, and an adult who consumes a daily intake of one fruit for one year is equivalent to the amount of annonacin injected into rats, which induced the brain lesions [239]. It has been suggested that the toxicity might be related to the capacity of the tetrahydrofuran ring to chelate calcium ions [35]. Moreover, the fruit of *A. squamosa* has been analysed for its quantity of squamocin using HPLC-MS and reported 13.5–36.4 mg of squamocin for each fruit, and that long-term consumption of *A. squamosa* fruit may be a risk factor in the development of neurodegenerative disorders [240]. Additionally, the use of a dietary supplement sold in the USA containing an extract of *A. muricata* has been found to exhibit neurotoxic effects in human neuron cultures [241].

The interactions of *Annona* species with other drugs have been reported in other studies; for instance, administration of capsules of *A. muricata* leaves in combination with glibenclamide resulted in improved glycaemic control compared to patients who received only glibenclamide [242]. An additional study reported that a combination of aqueous custard apple leaf extract and glipizide enabled a decrease in the dose of glipizide by up to half in rats with type-2 diabetes and reduced the risk of requiring insulin therapy [243]. These outcomes suggest the potential use of certain *Annona* species in conjunction with antidiabetic medications to maximize the efficacy of a lower therapeutic dose.

## 9. Conclusions

This review provides a comprehensive summary of the botanical features, ethnomedical uses, pharmacology and phytochemistry of the main species of Annonacae family and, in particular, the *Annona* genera used in traditional medicine practices. Of the many members of the Annonacae family, the *Annona* species is heavily used in traditional medicines across the world. Among the 30 reviewed *Annona* species, six species *A. squamosa, A. muricata, A. cherimola, A. senegalensis*, *A. reticulata* and *A. coriacea* are the most widely studied for their pharmacological activities and phytochemical profiles of their bark, leaves, fruits and seeds. Various pharmacological properties have been reported, including antidiabetic, hepatoprotective, anti-inflammatory, antiprotozoal, antitumor, antioxidant, antimicrobial and anticonvulsant activity.

With regard to the phytochemistry of *Annona* species, the main classes of constituents identified to date are acetogenins, alkaloids, phenols and essential oils. The alkaloids are mainly present in the leaves, whereas acetogenins are present in the seeds and found in smaller quantities in the pulp and leaves of *Annona* species. The chemical profiles of the acetogenins present in different species have been extensively studied and their anticancer activity investigated, with low concentrations exhibiting chemotoxicity against several cancer cell lines. These preclinical results, along with the reported case studies, suggest that further clinical studies evaluating the role of acetogenins in the treatment of various types of cancers are warranted. Importantly, formulations, including the parts of the *Annona* species used, agricultural practices and the extraction methods vary considerably, leading to likely variations in the phytochemical and pharmacological profiles. In this respect, further characterization of standardized formulations of *Annona* species is required to predict likely clinical effects. Additional interesting results on the antidiabetic effects of fruits from *Annona* species also warrant further investigation as nutraceuticals to assist in the therapy of diabetes.

## Figures and Tables

**Table 1:** Uses of most commonly used Annonaceae family in traditional medicines.

Annonaceae Species	Region	Local Name	Medicinal Uses	Part Used	Mode of Usage	References
*Alphonsea javanica* Scheff.	Indonesia	Aku Battu	Rheumatism and edema	Leave	Ethanolic extract	[14]
*Annickia chlorantha* (Oliv).	Cameroon	African yellow wood (c) Moambe Jaune	Treatment of sores Antipyretic Antiemetic Stimulant Tuberculosis Treatment of jaundice Urinary tract infection	Bark	Powder Crushed bark and drink extract Decoction Decoction in baths Decoction Decoction	[12,13]
*Annickia affinis* (Exell) Versteegh & Sosef	Cameroon	African yellow wood	Wound healing Antiemetic Antipyretic	Stem bark	Decoction of stem bark	[13]
*Anonidium floribundum* Pellegr	Cameroon	Eboum, Libanga Ebom	Poison antidote Dysentery Antipyretic	Roots Root/Bark Leaves	Decoction taken orally	[12,15]
*Anonidium mannii* (Oliv.)	Cameroon	Ebome; Npole Wapo’o, Ebome Afan	Antipyretic	Stem bark	Decoction of stem bark	[13]
*Boutiquea platypetala* (Engl.)	Cameroon	Not reported	To treat fresh wounds	Leaves	Pounded fresh leaves	[12]
*Cananga odorata* (Lam.) Hook and Thomson	Malaysia and India	Kenanga utan, Perfume tree, Cananga oil, Ylang ylang	Rheumatism Ophthalmic inflammation and wound healing	Bark	Bark extract eye drops for inflammation and decoction are used to wash fresh wounds	[11]
*Duguetia chrysocarpa* Maas	Brazil	Pindaíba-da-mata	Bowl and rheumatism inflammation	Leave and twigs	Leaves and twigs extract taken to relieve inflammation	[16]
*Enicosanthellum pulchrum* (King) Heusden	Malaysia	Disepalum	Rheumatism fever, edema and asthma	Leave	Decoction can be used for asthma and rheumatism	[17]
*Enantia chlorantha* var. soyauxii Engler and Diels	Africa	African yellow wood	Arthritis and wound healing	Bark	Powdered bark with citrus lemon used as dressing	[11]
*Friesodielsia enghiana* (Diels.) Verdc	Cameroon	Lonkosso	Analgesic	Bark	Decoction of bark is taken orally	[15]
*Friesodielsia gracilipes* (Benth.) Steenis	Cameroon	Ntonda	Treatment of sores, skin infection, ulcers, and jaundice	Bark and wood	Decoction of bark and wood	[12]
*Fissistigma oldhamii* (Hemsl.) Merr	Southern China	Oldhamii	Rheumatoid arthritis	Stems and roots	Powdered of stems and roots and orally ingested	[11]
*Greenwayodendron Suaveolens* (Engl and Diels) Verdc	Not reported	Otounga	Aphrodisiac and Vermifuge Rheumatic pains, fevers, headache, stomach-ache	Root Leaves and bark	Chew roots Pulverized leaves or bark and mixed with seeds of *Aframomum melegueta*	[15]
*Isolona hexaloba* (Pierre) Engl & Diels	Democratic Republic of Congo	Bodzungu	Malaria	Stem bark	Decoction of stem bark	[18]
*Monodora myristica* (Gaertn.) Dunal	Ivory coast	M Kpo. Abidjan district	Eye diseases and hemorrhoids, febrile pains and headache	Fruits Seed	Fruits and seeds consumed whole or ground to be used in soup and stews	[19]
*Monodora tenuifolia* Benth	Not reported	African nutmeg Ebom osoé Grandes feuilles	Toothache Dysentery and fevers	Root Bark and root	Clean the roots, boil and rinse the mouth Prepared as a decoction and used as an enema	[12]
*Polyalthia suaveolens* Engl and Diels	Cameroon	Diels; Otungui; Ntounga	Analgesic, Antiepileptic Antipyretic Treatment of jaundice	Stem bark	Decoction of stem bark	[13]
*Polyalthia longifolia* (Sonn.) Thwaites	India	Ashoka	Fever	Bark	Decoction of bark	[20]
*Xylopia aethiopica* (Dunal) A.Rich	Sudan	Ethiopia or Negro pepper	Rheumatism, colic pain, headache, and neuralgia	Fruits	Ethanolic fruit extract or dried fruits are used as whole	[21]
*Xylopia aromatic* Lam. Mart	Columbia	Monkey pepper	Pulmonary inflammation and hemorrhoids	Roots Leaves	Insertion of root pieces into rectum and leaves burnt and smoke inhaled	[22]
*Xylopia parvifolia* Hook.f. and Thomson	East and Central Africa, India	Netawu/Athu ketiya	Gastrointestinal ulcers Analgesic	Roots	Decoction Finely dried powder	[23]
*Xylopia staudtii* Engl & Diels	Not reported	Ntom, Odjobi Bush pepper (c)	Cold and headache treatment	Bark	Powder	[12]
*Monodora tenuifolia* Benth	Cameroon	Ebome osso	Joint and muscle pain, promotion of breast milk production and headache	Stem bark	Decoction of stem bark powder	[13]
*Uvaria* acuminata Oliv	Cameroon	Nosonaback	Typhoid and Yellow fever Headache and epilepsy	Stem bark	Decoction of stem bark	[13]

**Table 2:** Representative phytochemicals isolated from plants of Annonaceae.

Species	Part	Compounds	Class	References
*Anaxagoma dolichocarpa* Sprague and Sandwith	Fruits	*p*-Cymene Spathulenol Caryophyllene oxide Guaiene	ESO	[5]
*Anomianthus dulcis* (Dunal) J. Sinclair	Stem	(−)-Anolobine (−)-Anonaine	ALK	[24]
*Artabotrys pierreanus* Engl. & Diels	Stem bark	Cyperene Caryophyllene oxide Cyperermone Cadalene	ESO	[5]
Artabotrys hexapetalus (L.f.) Bhandari	Aerial parts	9-Oxo-asimicinone Artapetalin-A Artapetalin-B	ACT	[25,26]
*Goniothalamus giganteus* Hook.f. & Thomson	Bark	Pyranicin Pyragonicin Goniotrionin	ACT	[27]
*Miliusa balansae* Finet & Gagnep	Leaves and branches	Ombuine Chrysosplenol Pachypodol Chrysosplenol C	FLA	[28]

ALK (Alkaloids), ACT (Acetogenins), ESO (Essential oils) and FLA (Flavonoids).

**Table 3:** Pharmacological activities of some isolated compounds from Annonaceae species.

Species	Part Used	Isolated Compounds	Pharmacological Activity	Mechanism of Action	References
*Alphonsea javanica* Scheff	Leaves	(+)-Altholactone (+)-Goniothalmin	Anti-inflammatory	Inhibited lipopolysaccharide (LPS) induced NO production in RAW 264.7 macrophages with IC_50_ = 0.8 µM.	[14]
*Artabotrys hexapetalus* (L.f.) Bhandari	Roots, stems, and leaves	Artabonatine B Squamolone	Anticancer	Exhibited activity against 2,2,15 and Hep G2 cell lines with IC_50_ 11.0 and 9.1 µg/mL. Displayed activity against Hep G2 cell lines with IC_50_ 2.8 µg/mL.	[29]
*Cananga odorata* (Lam.) Hook.f. & Thomson	Fruits	Cleistopholine	Cytotoxic	Exhibited cytotoxicity against both Hep 2,2,15 and Hep G2 cell lines with IC_50_ 0.54 and 0.22 µg/mL, respectively.	[30]
*Goniothalamus tamirensis* Pierre ex Finet & Gagnep	Stem bark	Dielsiquinone	Cytotoxic	Displayed cytotoxic activity against U251, RPMI, MCF7, HT029 and A549. with ED_50_ 0.37, 0.11, 0.11 1.12 and 0.11, respectively.	[31]
*Guatteria blepharophylla* Mart	Bark	Isocoreximine	Anti-proliferative activity	Exhibited activity against UACC-62, NCI-H460, HT-29 and MCF-7 with TGI > 764.52 µM.	[32]
*Rollinia sylvatica* A.St.-Hil	Leaves	Hinesol z-Caryophyllene beta-Maaliene	Anti-inflammatory	Leukocytes migration was significantly reduced at concentrations of 36.04–45.37 µg/mL.	[33]

**Table 4:** Botanical information of some *Annona* species.

Species	Synonyms	Local Names	Geographic Distribution	References
*A. cherimola*	*A. tripetala* Aiton *A. pubescens* Salisb	Chirimoya Chirimolia Cerimoya Cherimoyer Momona	South Africa, China Egypt Eritrea Myanmar Philippines India France Italy Mexico, Ecuador Portugal Peru	[6,48]
*A. coriacea*	*A. coriacea* var. amplexicaulis S.Moore, *A. coriacea* var. cuneate, *A. coriacea* var. pygmaea Warm	Marolo Araticum Marolino	Brazil (Cerrado, Caatinga)	[10,49]
*A. cornifolia*	*A. walkeri* S. Moore	Araticum-mirim	Brazil	[50]
*A. crassiflora*	*A. macrocarpa* Barb *A. rodriguesii* Barb	Araticum Pinha-docerrado Cerrado pinecone Marolo Cabeça de negro	Brazil	[51]
*A. macroprophyllata*	*A. diversifolia* Saff	Ilama, Papauce Anona blanca	Mexico China India	[52,53]
*A. montana* Macfad	*A. Montana* f. marcgravii (Mart.) Porto	Mountain soursop False graviola jacá do Pará Araticum grande Shan di fan li zhi	Southern Asia, South America Amazon Rainforest and Atlantic Forest	[10]
*A. muricata*	*A. macrocarpa* Barb *A. muricata* Guanabanus *A. cearensis* Morales	Brazilian pawpaw Soursop, ci guo fan li zhi, Graviola Araticum grande Mullu Raama Phala, Corossol Catuche	Tropical regions of Americas Malaysia, Myanmar, Pakistan, India, Indonesia, China	[6,10]
*A. reticulata*	*A. excelsa* Kunt *A. laevis* Kunth *A. longifolia* Moc *A. riparia* Kunth	Custard apple Bullock’s heart	Indonesia, West Indies, Bangladesh, China, India	[54]
*A. sclerophylla* Saff	Sulcata Urb *A. spinescens* Mart	Not reported	Brazil	[10]
*A. senegalensis*	*A. senegalensis* var. arenaria Sillans *A. senegalensis* var. Bail. Wild *A. senegalensis* var. glabrescens Oliv $*A. senegalensis* var. cuneata Oliv *A. arenaria* Thonn *A. chrysophylla* *A. chrysophylla* var. porpetac Bail *A. porpetac* Bail	Wild soursop Sour soup Abo Uburuochaand Gwandar	Nigeria	[6]
*A. squamosa*	*A. asiatica* L. *A. squamosa* f. Parvifolia Kuntze *A. cinerea* Dunal *Guanabanus squamosus* (L.) M.Gómez	Custard apple Sweetsop Tiep baay Amritaphala Chirimoya Fruta do conde Squamosus Gomez Guanabanus	Egypt, Sudan, Pakistan, Thailand, China, India, Costa Rica,	[6,10]

**Table 5:** Traditional uses of common *Annona* species.

Species	Region	Local Name	Medicinal Uses	Part Used	Mode of Usage	References
*A. ambotay* Aubl	French Guiana	Not reported	Treating fever	Leaves and bark	Leaves and bark crushed and rubbed on body	[18]
*A. cherimola* Mill.	Tropical America Asia Gabon Cultivated in Spain and Australia	Cherimola Cherimoya Chirimoya Custard apple Mao ye fan li zhi	Abortion Anti-anxiety Cough Diarrhea Hypercholesterolemia Infections Painful inflammations Parasitic Sedative	Aerial parts Fruit Leaf Root Seed Stem	Not reported	[87,88]
*A. coriacea* Mart.	Brazilian (Cerrado, Caatinga)	Araticum Marolino Marolo	Anthelmintic Chronic diarrhea Inflammation Leishmaniasis Malaria Rheuma	Leaves Root Seeds	Not reported	[89]
*A. cornifolia* St-Hil	Bolivian and Brazilian savannah	Not reported	Antiulcerative (green fruit)	Seeds	Not reported	[90]
*A. crassiflora* Mart.	Brazil (Cerrado)	Araticum of the Cerrado Araticum-mirim Marolo Panã	Analgesic Antimicrobial Antirheumatic Carminative Digestive Rheumatism, Anti-inflammatoryWound healing	Leaves Root bark Root wood Seeds Fruit	Not reported	[43,91,92]
*A. cuneata* (Oliv.) R.E. Fr	Congo	Not reported	Asthenia Female sterility Hernia Parasitic infections Venereal diseases	Root bark Stem bark	Not reported	[93]
*A. dioica* A. St.-Hil.	Brazil (Cerrado, Pantanal)	Ceraticum and ariticum	Diarrhea Rheumatism	Fruits Leaves	Dried leave paste and fresh fruit decoction	[11,33]
*A. diversifolia* Saff	Tropical forest of Central America China	Ilama Papausa White anona, Yi ye fan li zhi	Arthritic pain Anti-spasmodic	Leaves Seeds	Not reported	[40,53]
*A. foetida* Mart	Brazil	Araticum-da-caatinga	Malaria	Bark and leaves	Decoction of bark and leaf	[94]
*A. glabra* L	Caribbean	Mamain	Fever	Leaves	Not reported	[95]
*A. glauca* Schumach. & Thonn	West Tropical Africa (Senegal, Ghana, Suriname)	Dangan Mampihege, Mandé sunsun Tangasu	Arachnicides Blennorrhoea Diuretic Fish-poisons Insecticides	Roots Seeds	Not reported	[96]
*A. haematantha* Miq	French Guiana South American tropical rainforest	Not reported	Fever	Leaves Bark Roots	Leaves and bark crushed rubbed on body	[18,97]
*A. montana* Macfad.	South America Southern Asia The Amazon Brazil (Mata Atlántica, Pantanal)	Mountain soursop Shan Di fan li zhi false Graviola Araticum grande Jacá do Pará	Against snake bite Against obesity	Leaf Pulp juice Seed Stem Twig	Not reported	[98,99]
*A. muricata* Linn	Brazil	Araticum Condessa Graviola	Anthelmintic Analgesic, neuralgia, rheumatism, arthritis pain	Fruit, juice, and crushed seeds Fruit and leaves	Juice of fruit Water extraction of the leaf	[66]
*A. pickelii* (Diels) H. Rainer	Mexico Caribbean Central America Venezuela Colombia BelizeCentral America South America Southern Asia Africa Madagascar	Sincollo Soncoyo Bullock’s-heart Custard apple Anona blanca Anona Niiu xin fan li zhi	Contraceptive Blood dysentery Cold Stomachache Fainting spinal disorders Fever Hysteria Influenza Mental depression Skin diseases Unhealthy ulcers Wounds	Leaf Leaf Root Stem Seed Aerial parts Bark Fruit Leaf Root Seed Stem bark	Not reported	[100,101]
*A. reticulata* Linn	West Indies	Ramphal	Bronchitis Asthma Bowel inflammation	Fruit Seeds Leaves	Decoction of fruit Oral ingestion of powdered seeds Oral ingestion of the leaf powder	[102]
*A. senegalensis* Persoon	Nigeria	Ukopko (Idoma)	Anti-inflammatory Analgesic Anthelmintic Cancer Diarrhea Epilepsy Infectious diseases Inflammations Sleeping sickness Snakebite Cardiovascular diseases Diabetes Febrile seizures Gout Mental disorders Painful	Leaves Seed Stem Bark Root bark	Roots and bark are ground together and their decoction is used	[103]
*A. squamosa* Linn	Cameroon	Sugar apple (English); Kedahan (Yambetta)	Vomiting, abscesses, muscle aches, fever, and skin disease	Leaves	Decoction of leaves	[13]
*A. vepretorum* Mart	Brazil	Araticum Bruteira	Analgesic and anti-inflammatory	Leaves	Methanolic leaf extract	[104]

**Table 6:** Compounds isolated from plants of *Annona* genus.

Species	Part	Isolated Compounds	References
*A. amazonica* R.E. Fries	Stems	Cassythicine Liriodenine (ALK)	[110]
*A. cherimola*	Root	Corytenchine, Isocoreximine (ALK)	[111,112,113,114,115,116]
	Fruit	α-Pinene, α-Thujene, Terpinen-4-ol, Germacrene D (ESO)	
	Seed	2,4-*cis*-Annocherinones, Annocherin, 2,4-*trans*-Isoannonacins, Annocherimolin, Annomolin, Annomocherin, Annomontacin, Annonacin, Asimicin, Tucumanin, 2,4-*trans*-Annocherinones, 2,4-*cis*-Isoannonacins, *cis*-Annonacin, Annogalene, Annosenegalin, Annomolon A, Annomolon B, Cherimolacyclopeptide C (ACT)	
	Stem	Annocherine A and B, Artabonatine B, Romucosine H, Cherianoine (ALK)	
*A. coriacea* Mart.	Bulb	Crolechinic acid, Crolechinic acid (methyl ester), Annonene, Annonalide (ESO)	[117,118,119,120,121,122,123]
	Seed	Gigantecin, Coriapentocin A and B, Bullacin (ACT)	
	Leaf	Quercetin-3-*O*-β-(6″-*O*-β-glucosyl)-glucoside, Quercetin-3-*O*-β-(6″-*O*-α-rhamnosyl)-galactoside, Trigonelline, Rutin, Hyperin, Hyperin, Isorhamnetin-3-*O*-β-glucoside, Isorhamnetin-3-*O*-β-galactoside, Isoquercitrin, Isoquercitrin, Nicotiflorin, Biorobin, Keioside, Cacticin, Isorhamnetin-3-*O*-β-glucoside, Narcissin, Rutin (FLA)	
	Root	Coriacin, Coriadienin, Coriaheptocin A and B, Coriacyclodienin, Coriacycloenin, 4-Deoxycoriacin, Annoheptocin A and B (ACT)	
*A. crassiflora*	Leaf	Kaempferol-3-*O*-β-diglucoside, Kaempferol-3-*O*-β-glucoside, Quercetin-3-*O*-β-D-galactopyranoside, Epicatechin, Quercetin-3-*O*-β-L-arabinopiranoside (FLA)	[124]
*A. foetida*	Bark	Annomontine, *N*-Hydroxyannomontine, Liriodenine, *O*-methylmoschatoline (ALK)	[125,126,127]
	Leaf	(*E*)-caryophyllene, Bicyclogermacrene, α-Copaene (ESO)	
	Branch	Atherospermidine (ALK)	
*A. glabra*	Fruit	16α-17-Dihydroxy-ent-kauran-19-oic acid, 16α-Hydro-ent-kauran-17-oic acid, 16β-Hydroxy-17-acetoxy-ent-kauran-19-oic acid, 16α-Hydro-19-al-ent-kauran-17-oic acid, 16β-Hydro-ent-kauran-17-oic acid, 19-nor-ent-Kauran-4α-ol-17-oic acid, Annoglabasin A and B, ent-Kaur-15-ene-17,19-diol, ent-Kaur-16-en-19-ol, ent-Kaur-16-en-19-oic acid, Methyl-16α-hydro-19-al-ent-kauran-17-oate (ALK)	[128,129,130,131,132]
	Fruit & stem	Annoglabasin A, B, C, D, E and F, (−)-Anonaine, (−)-Asimilobine, (−)-Kikemanine, (−)-Nornuciferine, (+)-Stepharine, Blumenol A, Liriodenine, *N*-*p*-Coumaroyltyramine, (−)-*N*-Formylanonaine, (+)-Nordomesticine, Annobraine, Dehydrocorydalmine, Lysicamine, *N*-trans-Feruloyltyramine (ALK), 6-*O*-Palmitoyl-β-sitosteryl-D-glucoside, β-Sitosteryl-glucoside, Stigmasteryl-D-glucoside, β-Sitosterol, Stigmasterol (STE)	
	Seed	Isodesacetyluvaricin (ACT)	
	Leaf	Bullatanocin, Glabracins A and B, Javoricin, Glacins A and B (ACT), 3-*O*-α-L-Arabinopyranoside, 3-*O*-β-D-Glucopyranoside (GLU) (–)-Actinodaphnine, (−)-Asimilobine, (−)-Anolobine, (−)-*N*-Methylactinodaphnine, (−)-Roemeroline, (+)-Boldine, (+)-Norisodomesticine, (+)-Stepharine, Liriodenine, (−)-Pallidine, (+)-1S,2S-Reticuline *N*-oxide, (+)-Magnoflorine, (+)-Reticuline (ALK) Quercetin, Quercetin−3-*O*-β-D-galactopyranoside (FLA)	
*Annona leptopetala* (R.E.Fr.) H. Rainer	Leaves and branches	Laurotetanine, Nornuciferine, Corypalmine, NorannuradhapurineAnonaine (ALK)	[133]
*A. montana*	Leaf	Annolatine, Annoretine, Liriodenine, Argentinine (ALK), β-Sitosterol-β-D-glucoside, β-Sitosterol (STE), Montanacin-K, L, C, D, B and E, Annonacin-10-one, Annonacin-A, *cis*-Annonacin-10-one, Annonacin, *cis*-Annonacin (ACT)	[134,135,136,137]
	Seeds	Montalicins G, Montalicins H Monlicins A & B, Murisolin, 4-Deoxyannomontacin, Muricatacin (ACT)	
	Stem	*N-trans*-Feruloyltyramine, *N-p*-Coumaroyltyramine, *N-trans*-Caffeoyltyramine (PHE)	
*A. muricata*	Seed	2,4-*cis*-Gigantetrocinone, 2,4-*trans*-Isoaiinonacin, 2,4-*trans*-Gigantetrocinone, 2,4-*trans*-Isoannonacin-10-one, Gigantetrocin-A, Muricatenol, Annomontacin, Gigantetronenin, Annonacin A, Annoreticum-9-one, *cis*-Annomontacin, Murisolin, Muricin H, Xylomaticin, Muricin I, *cis*-Annonacin, *cis*-Goniothalamicin, *cis*-Annonacin-10-one, Arianacin, Javoricin, Donhexocin, Murihexol, Cohibins C, Cohibins D, Gigantetrocin B, Longifolicin, Muricin A, B, C, D, E, F and G, Annomuricatin B and C (ACT)	
	Stem bark	Muricatin A, B and C (ACT)	
	Fruit	Epomuricenins-A and B, Epomurinins-A and B, Epomusenins-A and B, Muricin J, K and L (ACT) Asimilobine, Nomuciferine, Annonaine (ALK)	[138,139,140,141,142,143,144,145,146]
	Fruit & Root	Sabadelin (ACT)	
	Leaf	Annonacin, Annomuricin C, Muricatocin C, (2,4-*cis*)-10*R*-annonacin-A-one, (2,4-*trans*)-10*R*-annonacin-A-one, Annohexocin, Annomutacin, Annopentocins A, B and C, Annomuricine, Muricapentocin, Annomuricins A and B, *cis*-annomuricin-D-ones, trans-annomuricin-D-ones, Muricatocins A and B, Murihexocin A and B, Muricoreacin, Murihexocin C (ACT) (*R*)-4-*O*-methylcoclaurine, (*R*)-*O*, *O*-dimethylcoclaurine, (*R*)-Anonaine, Annonamine, (*S*)-Norcorydine, Anonaine, Isolaureline, Xylopine (ALK) Catechine, Epicatechine, Gallic acid, Chlorogenic acid, Kaempferol, Kaempferol-3-*O*-rutinoside, Quercetin-3-*O*-rutinoside, Quercetin-3-*O*-glucoside, Quercetin-3-*O*-neohispredoside, Quercetin-3-*O*-robinoside, Annoionols A and B, Annoionoside (FLA)	
	Leaf & seed	Annonacin, Annocatacin A and B, Annonacinone, Annocatalin, cis-Corossolone, Goniothalamicin, Isoannonacin, Corossolone (ACT)	
	Pericarp	Annonacin, Annonacin A, Annomuricin A (ACT)	
	Root	Annonacin, Muridienins-1, 2,3 and 4, Chatenaytrienins-1, 2 and 3, Muricadienin, Montecristin, *cis*-Panatellin, *cis*-Reticulatacin-10-one, *cis*-Uvariamicin IV, Coronin, *cis*-reticulatacin, *cis*-Solamin, Cohibins A and B (ACT)	
*A. purpurea*	Leaf	Lirinidine, 7-Formyl-dehydrothalicsimidine, 7-Hydroxy-dehydrothalicsimidine, *N*-Methylasimilobine, *N*-Methyllaurotetanine, Thalicsimidine, Norpurpureine (ALK)	[105,147]
	Root	Annomontine (ALK)	
*A. reticlata*	Leaf	Dopamine, Salsolinol, Spathenelol, Muurolene, Coclaurine, Copaene, Eudesmol (ESO), Squamone, Solamin, Rolliniastatin 2, Annoreticuin-9-one, Annomonicin, Annonaretin A (ACT)	[54,148,149,150,151,152]
	Stem bark	Dopamine, Salsolinol (ESO), Reticullacinone, Rolliniastatin-2, (ACT)	
	Root	Liriodenine, Norushinsunine, Neoannonin, Reticuline (ALK) Spathenelol, Copaene, Eudesmol, Muurolene (ESO)	
	Bark	Reticulatacin, Liriodenine, Copaene, Coclaurine (ALK) Patchoulane (ESO) Molvizarin, Bullatacin (ACT)	
	Seed	Squamocin, *cis*-/*trans*-isomurisolenin, Bullatacin, *cis*-/*trans*-Bullatacinone, Annoreticuin, Annoreticuin-9-one, Solamin, Annomonicin, Isoannonareticin, Rolliniastatin-1, 2 Squamone, Annonareticin, 2, 4-*cis*-Isoannonareticin, Solamin, Murisolin, Reticulacinone, Annomonicin, Sitosterol, Annoreticuin (ACT), Myrcene, Limonene, Germacrene D (ESO)	
	Fruit	Terpinen-4-ol, Germacrene D, Limonene, Pinene, Myrcene (ESO)	
	Root bark	Anonaine, Michelalbine, Reticuline, Oxoushinsunine (ALK)	
*A. senegalensis*	Leaf	(−)-Roemerine, α-Humulene, γ-Cadinene, Germacrene D, β-Caryophyllene (ESO)	[153,154]
	Aerial parts	(−)-Anonaine, (−)-Asimilobine, (+)-Nornantenine (ALK) (+)-Catechin (FLA)	
	Seed	Annogalene, Annosenegalin (ACT)	
*Annona sericea* Dunal	Leaf	Nornantenine, Nornuciferine, Isoboldine, Lysicamine, Hydroxynornuciferine (ALK	[155]
*A. squamosa*	Leaf	(−) Anonaine, O-Methylarmepavine, β-Caryophyllene, β-Cedrene, (E)-Caryophyllene, Germacrene D, Bicyclogermacrene, Quercetin-3-*O*-glucoside (ESO)	[156,157]
	Bark	2,4-*cis*-Mosinone A, 2,4-*trans*-Mosinone A, Annoreticum-9-one, Mosin B and C, Bullatacin, Bullatacinone, Squamone (ACT)	
	Pulp fruit	α-Pinene, Limonene, Sabinene (ESO)	
	Stem	11 *ent*-Kauranes, 10-nor-*ent*-Kaurane-4α,16β,17-triol, 16α,17-Dihydroxy-*ent*-kauran-19-oic acid, 16β,17-Dihydroxy-*ent*-kauran-19-al, 16β-Hydro-*ent*-kauran-17,19-dioic acid, 17-Hydroxy-16β-*ent*-kauran-19 oic acid, *ent*-Kaur-16-en-19-oic acid, 16α,17-Dihydroxy-*ent*-kauran-19-al, 16α-Hydro-19-al-*ent*-kauran-17-oic acid, 16β,17-Dihydroxy-*ent*-kauran-19-oic acid, 16β-Hydroxy-17-acetoxy-*ent*-kauran-19-oic acid, 4α-Hydroxy-19-nor-*ent*-kauran-17-oic acid (ALK)	
	Seed	Neoannonin-B, Annosquamins A, B and C, Annosquacin-I, Annosquamin A, B and C, Annosquatin A and B, Annotemoyin-1 and 2, Cherimolin-1 and 2, Diepomuricanin A and B, Dieporeticenin, Dieposabadelin, Squadiolin A, B and C, D, E, F, G, H, I, J, K, L, M and N, Squamostanin A, B, C, D, E and F, Cyclosquamosin A, B, C, D, E, F, G, H and I, Squamin A and B (ACT)	
*A. vepretorum*	Leaf	Spathulenol, Bicyclogermacrene, α-Phellandrene (ESO)	[116,158]

ALK (Alkaloids), ACT (Acetogenins), ESO (Essential oils). STE (Sterols), FLA (Flavonoids), PHE (Phenolics).

**Table 7:** Pharmacological activities of *Annona* species.

Species	Biogeographical Distribution	Used Part	Traditional Use	Pharmacological Activities	Extract/Compound Evaluated	References
*A. ambotay*	South American tropical rainforest	Trunkwood	Antipyretic	Antimicrobial	Alkaloids	[159]
*A. bullata*	Endemic of Cuba	Bark	Not reported	Antitumoral	32-Hydroxybullatacinone	[160]
*A. cherimola*	Tropical America, Asia, Spain Australia, Gabon	Aerial partsFruit Leaf Root Seed Stem	Abortion Anti-anxiety Cough Diarrhea Hypercholesterolemia Infections Painful inflammations Parasitic Sedative	Antidepressant Antifungal Antiprotozoal Antitumoral Antihypercholesterolemic Antiulcerative Antiviral Insecticidal Vasodilator	Acetogenins: Molvizarin, Squamocin, Cherimolin−1, Motrilin, Aherradurin, Tucumanin Annomocherin, Annonacin, Annomontacin, Alkaloids: Roemerine, Anonaine, Dehydroroemerine	[37,161]
*A. coriacea*	Brazilia (Cerrado and Caatinga)	Leaf Root Seed	Leishmaniasis Malaria Rheuma Anthelmintic Chronic diarrhea Inflammation	Antifungal Anti-inflammatory Antitumoral Insecticidal Leishmanicidal Trypanocidal	Acetogenins: Annoheptocins A-B, coriacin, 4-Deoxycoriacin, Coriaheptocins A-B, Coriadienin, Gigantecin	[10]
*A. muricata*	America, Asia, Africa	Bark Leaf Fruit Root Root bark Seed Stem bark	Anthelmintic Antiscorbutic Asthma Cancer Cough Cystitis Diabetes Diuretic	Anti-arthritic Antidepressant, Antidiabetic Anti-inflammatory Antimicrobial Antimalarial Antiviral Hepatoprotective	Acetogenins: Solamin, Muricin H, Muricin I, cis-Annonacin, Muricins A-G, Muricoreacin, Muricapentocin, Gigantetrocin A, Annopentocins A-C	[162,163]
*A. salzmannii*	Brazil	Bark Leaf	Not reported	Antioxidant Antimicrobial	Alkaloids: Reticuline, Anonaine, Laurelliptine, Isoboldine	[101]
*A. senegalensis*	Madagascar, Comoros, Cape Verde, Tropical Africa	Leaves Seed Stem Bark Root bark	Anti-inflammatory and Analgesic Anthelmintic Cancer Diarrhea Epilepsy Infectious diseases Inflammations Sleeping sickness Snakebite	Anticonvulsant Antidiabetic Antidiarrheal anthelmintic Anti-inflammatory Antimalarial Antimicrobial Antioxidant Insecticidal Hepatoprotective Antitumoral	Aqueous extract. Ethanolic extract Terpenoids, coumarins, flavonoids, tannins, alkaloids, quinones. Methanolic extract containing Annosenegalin, Annogalene.	[10,164]
*A. squamosa*	Tropical America, Asia Australia	Seed Stem Bark Root bark	Analgesic Anthelmintic Antirheumatic Cancer Digestive Headache Anti-inflammatory Antimicrobial Carminative	Antibacterial Antidiabetic Antilipidemic Antioxidant Antimalarial Antigenotoxicity Antileishmanial	Acetogenins: Squadiolins A and B, Squafosacin B, Bullatacinona, Squamona, Tetrahydrosquamone Monoterpenes: Limonene, β-Cubebene, β-Caryophyllene, Spathulenol, Caryophyllene oxide	[10]

**Table 8:** Antiamoebic activity of squamins C-F versus *Acanthamoeba* spp. Strains.

Compounds	*A. castellanii* Neff IC_50_ (μM)	*A. polyphaga* IC_50_ (μM)	*A. griffin* IC_50_ (μM)	*A. quina* IC_50_ (μM)
Squamin C	20.77 ± 3.48	71.78 ± 0.41	38.81 ± 7.34	24.28 ± 0.64
Squamin D	18.38 ± 1.14	71.57 ± 0.14	39.53 ± 5.90	26.52 ± 0.87
Squamin E	21.00 ± 0.86	62.19 ± 15.52	44.75 ± 2.06	25.82 ± 0.99
Squamin F	18.02 ± 3.28	64.08 ± 12.42	50.49 ± 6.92	30.32 ± 0.27

**Table 9:** Anticancer activity of isolated compounds from *Annona* species and their mode of action.

*Annona* Species	Plant Part	Isolated Components	Cell Line or Animal Model	Mechanism of Action	References
*A. cherimola*	Seeds	Annomolin and Annocherimolin	Prostate tumor cell line (PC-3), breast (MCF- 7) and colon (HT-29) cancer cell lines	Exhibited potent cytotoxicity	[209]
	Leaves	Asimilobine	Acute myeloid leukemia cell line	Upregulation of Bax, downregulation of Bcl2, and cleavage of PARP	[210]
*A. crassiflor*	Crude extract	Catechin	Cervical cancer cell	Apoptosis via intrinsic pathway	[211]
*A. glabra*	Fruits	Annoglabasin H	Lung adenocarcinoma cell line (LU-1), human breast carcinoma (MCF-7), human melanoma (SK-Mel2)	Exhibited significant cytotoxic activity	[212]
		Annoglabayin	Human liver cancer cell line (Hep G2)	Apoptosis via mitochondrial pathway	[132]
		Cunabic acid and ent-kauran-19-al-17-oic acid	Liver cancer (HLC) cell line SMMC-7721	Apoptosis via down-regulation of BCL-2 gene and upregulation of bax gene	[213]
	Leaves	Asinicin	Human monocytic leukemia cells (CRL-12253)	Mitochondria mediated anticancer and antiproliferative effects	[214]
		Annoglacin A and B	Human breast carcinoma (MCF-7) and Pancreatic carcinoma (PACA-2) cell lines	Suppressed proliferation	[215]
		Icariside D2	Human leukemia cell line (HL-60)	Induced apoptosis and decreased phosphorylation of AKT in cells	[216]
*A. muricata*	Leaves	Annomuricin	Breast cancer cell	Suppressed breast cancer proliferation and induced apoptosis	[217]
		Muricoreacin, Murihexocin	Colon cancer cell (HT-29, HCT-116)	Up-regulation of Bax, downregulation of Bcl-2 proteins and activated initiator and executioner caspases	[218]
		Annomuricine, Muricapentocin	Pancreatic carcinoma (PACA-2) and colon adenocarcinoma (HT-29) cell	Exhibited repressive effect	[219]
		Muricatocins A and B	Lung tumor cell line (A-549)	Enhanced cytotoxic activity	[146]
	Fruits	Muricin M and Muricin N	Prostate cancer (PC-3) cells	Exhibited cytotoxicity	[220]
*A. purpurea*	Roots	Annopurpurici-ns A–D	HeLa and HepG2 cells	Mitochondrial membrane depolarization and apoptosis	[221]
*A. reticulata*	Fruits	Catechin	Breast cancer cell line (MCF-7)	Inhibition via apoptosis	[222]
	Seeds	Annonacin	T24 bladder cancer cells	Bax expression was induced, caspase-3 activity enhanced and caused apoptosis	[172]
		Bullatacin	Leukemia cell line (K562) and breast cancer cell line (MCF-7)	Cell death via apoptosis	[223]
	Leaves	Annomonicin	Colon cancer (HCT15), human lung cancer (Hop65) and human hepatoma (HEPG2) cell lines	Exhibited cytotoxic effect	[224]
		Rolliniastatin	Breast cancer cell (T-47D)	Caspases dependent apoptosis	[225]
*A. senegalensis*	Leaves	(−) Roemerine	Breast cancer MDA-MB-231 cells	Exhibited dose-dependent cytotoxicity via targeting the ribosomal protein eL42 and arresting the crosslinking reaction with tRNAox	[226]
	Bark	Kaurenoic acid	Pancreatic tumor (PANC-1) cell lines and Henrietta Lacks’ cervical cancer cell line (HeLa)	Exhibited significant cytotoxic activity	[227]
	Stem	Ent-kaurenoids	Breast cancer (MCF-7) cells, prostate cancer (PC-3) cells	Exhibited significant cytotoxic activity	[228]
*A. squamosa*	Leaves	Annoreticuin	Breast cancer cell (MCF-7)	Induced Apoptosis	[229]
	Seeds	Dieporeticenin B, Squamocin, Annosquatin III	Nasopharyngeal cancer (KB) cells, breast cancer (MCF-7) cells	Exerted inhibitory activity	[230]
		Asimilobine	Human colon cancer cell (WiDr)	Increased expression of caspase-3	[231]
		Annosquatin A, B	Human leukemia cell line (K-562), human colon carcinoma (COLO-205)	Reduced intracellular glutathione levels and regulation of Bcl-2 and PS externalization	[232]
		Annosquacins A-D, Annosquatin A, B	Human breast cancer cell line (MCF-7), human lung adenocarcinoma cell line (A-549)	Exhibited cytotoxic activity	[233]
		(−)-Anonaine	H22 solid tumor cell	Inhibition of IL-6/Jak/Stat3 pathway	[234]
	Bark	Coclaurine	DMBA painted hamsters	Enhanced lipid peroxidation	[235]
	Fruits	(−)-Ent-kaur-16-en-19 oic acid, 16α,17 dihydroxy-ent-kauran-19-oic acid	Dalton’s lymphoma cells, HeLa cells	Exhibited cytotoxic activity	[236]
*A. sylvatica* A.St.-Hil	Leaves	Quercetin Kaempferol	Anti-inflammatory	Leukocytes migration was significantly reduced at IC_50_ 8.53 and 10.57 µg/mL, respectively.	[189]
*A. vepretorum* Mart.	Leaves	Bicyclogermacrene	Antimicrobial	Against Candida tropicalis with a MIC value of 100 µg ·mL^−1^.	[237]

## Data Availability

No new data were created or analyzed in this study. Data sharing is not applicable to this article.
